# Rotation of the hammer and performance in hammer throwing

**DOI:** 10.3389/fbioe.2024.1449465

**Published:** 2024-08-27

**Authors:** Jiaru Huang, Leonardo A. Peyré-Tartaruga, Junlei Lin, Yu Shi, Wei Li

**Affiliations:** ^1^ School of Strength and Conditioning Training, Beijing Sport University, Beijing, China; ^2^ Human Locomotion Laboratory (LocoLab), Department of Public Health, Experimental Medicine and Forensic Sciences, University of Pavia, Pavia, Italy; ^3^ LaBiodin Biodynamics Laboratory, Escola de Educação Física, Fisioterapia e Dança, Universidade Federal do Rio Grande do Sul, Porto Alegre, Brazil

**Keywords:** female, velocity increment, rotation radius, horizontal azimuth angle, duration

## Abstract

**Objective:**

The purpose of this study was to determine the effects of hammer rotation on performance in hammer throwing.

**Methods:**

The hammer’s velocity increment at different stages, the duration of rotations at different phases, and the horizontal azimuth angle and rotation radius at critical instants were calculated and compared between the long and short trials for 26 female athletes in actual competitions.

**Results:**

Compared to short trials, female throwers’ long trials exhibited significantly larger release velocity (*p* < 0.001, ES = 1.42), greater velocity increment during the double support phase (*p* = 0.006, ES = 0.59), shorter duration during the single support phase (*p* ≤ 0.043, ES = 0.42–0.83), lower horizontal azimuth angle (*p* ≤ 0.027, ES = 0.46–0.57), and longer rotational radius at critical instants (*p* ≤ 0.021, ES = 0.48–0.73).

**Conclusion:**

During the process from the hammer head’s low point to high point, athletes should focus on increasing the rotation radius of the hammer head and accelerating the right foot’s landing speed during the single support phase. This approach aims to reduce the hammer’s horizontal azimuth angle at the right foot touchdown, enhance the acceleration performance during the double support phase, and increase the release speed.

## Introduction

Hammer throwing is one of the four throwing events in track and field. Hammer throwers throw a hammer weighing 7.26 kg and 121.5 cm in length for men or 4 kg and 119.5 cm for women at a high speed in a throwing circle that is 7 feet in diameter (Murofushi et al., 2017). The throwing movement can be divided into three stages: the preliminary winds, the turns, and the delivery (Murofushi et al., 2017). In the preliminary winds, the athlete stands at the back edge of the throwing circle, facing away from the throwing direction, and rotates the hammer 2–3 times around the body with both feet in contact with the ground. Then, the athlete enters the phase of the turn, pivoting on one foot’s heel and ball, continuously rotating and moving rapidly toward the throwing direction, creating a combined translational and rotational motion (Maheras, 2018; Murofushi et al., 2005). For an athlete rotating to the left,the left foot maintains contact with the ground throughout the rotation, while the right foot alternates between leaving and touching the ground, forming double support and single support phases in each turn (Castaldi et al., 2022). When the athlete reaches the front edge of the throwing circle, they forcefully release the hammer, completing the throw (Dapena, 1984; Dapena, 1986; Dapena and Feltner, 1989; Otto, 1992).

The release velocity, which is the sum of the velocity increments from the three stages (preliminary winds, turns, and delivery), is the primary factor affecting the throwing distance, with a high linear correlation between the two (Knudson, 2007; Ruiz and Dávila, 2009). To maximize the release speed, athletes must be able to continuously accelerate the hammer during the turns and delivery phases following the completion of the preliminary winds. In each turn, the hammer speed exhibits a periodic fluctuation, increasing during the double support phases and decreasing during the single support phases, but generally trending upward (Brice et al., 2015; Brice et al., 2018; Murofushi et al., 2007). Compared to the single support phase, the athlete maintains a more stable body posture during the double support phase, which is more conducive to increasing the hammer speed (Bartonietz, 2000; Brice et al., 2008). Therefore, many coaches and scholars recommend that throwers prolong the double support duration by shortening the single support phase (Bartonietz, 2000; Otto, 1992). To effectively shorten the single support (SS) phase duration and extend the double support (DS) phase duration, athletes need to plant their right foot earlier (Hay, 1978). This helps extend the DS phase but also aids in forming a “wound-up position,” where both feet’ axes lead the hips axis, the hips axis leads the shoulder axis, and the hammer lags far behind these three, thus facilitating greater hammer acceleration (Hay, 1978). The radius of rotation of the hammer is another critical factor influencing changes in hammer speed. According to the equation v=ω⋅r , theoretically, maintaining a longer radius is conducive to increasing the hammer speed, because a longer radius allows the thrower-hammer system to have a slower angular velocity at any given linear speed of the hammer (Dapena and Feltner, 1989). In that situation, muscles can exert greater forces at slower speeds, which is expected to facilitate an increase in the system’s angular momentum (Hill, 1922). However, as the rotation progresses, the rotation radius of the hammer tends to decrease, which is closely related to changes in the athlete’s posture (Dapena, 1986).

Scientific research on the hammer throw is limited, and the few existing studies have small sample sizes, with some findings still needing clarification (Brice et al., 2018; Castaldi et al., 2022). The hammer velocity curve shows that the double-support phase is the primary phase for speed increase in turn, this does not necessarily mean that the differences in release velocity at varying throwing distances are mainly derived from this phase. Identifying the specific phases that lead to differences in performance can provide more targeted guidance for athletes’ training practices. The notion that extending the duration of the double support phase is more beneficial for improving throwing performance has been questioned (Dapena, 1984; Dapena, 1989; Hirose et al., 2016). In fact, some high-level athletes have achieved longer throwing distances with significantly shorter double support phases (Bartonietz, 2000; Maheras, 2010). Additionally, while a longer rotation radius is generally more conducive to improving throwing performance, the hammer’s rotation radius fluctuates during each turn, and previous studies have yet to clearly identify the specific phases that lead to differences in performance. Moreover, due to variations in athletes’ arm lengths, directly comparing the radius graphs of different throwers may not be reasonable (Dapena and Mcdonald, 1989). While previous studies have advanced our understanding of hammer throw techniques, variations in athletes’ technical skills, body morphology, and physical fitness make it difficult to establish definitive guidelines for adjusting these factors to achieve the best performance. Therefore, we can only accurately identify and prescribe the adjustments needed to optimize hammer-throwing performance by comprehending the interference of these variable factors.

This study was to determine the effects of hammer rotation on performance in hammer throwing. It was hypothesized that 1) Compared to trials with the shortest official distances, the speed increment during the double support phase is greater in trials with the longest official distances. 2) Compared to trials with the shortest official distances, the duration of the double support phase is longer, and the duration of the single support phase is shorter in trials with the longest official distances. 3) Compared to trials with the shortest official distances, the horizontal azimuth of the hammer is smaller at critical instants in trials with the longest official distances among elite hammer throwers. 4) Compared to trials with the shortest official distances, the radius of rotation of the hammer is longer at critical instants in trials with the longest official distances among elite hammer throwers.

## Methods

### Participants

Comparisons of kinematic variables between the trials with the longest and shortest official distances were conducted for each participant in the same competition. This study analyzed the two trials with the greatest distance difference for the same athlete in the same competition from national competitions held in 2022, 2023, and 2024. If an athlete participated in multiple competitions, the competition with the greatest difference between their longest and shortest throws was selected. From 11 national competitions, after excluding trials with poor video quality caused by lighting and weather conditions and those where the hammer hit the net after release, a total of 26 athletes were chosen for the study. All athletes rotated to the left with the left foot keeping contact with the ground, and completed four turns.

### Data collection

**FIGURE 1:**
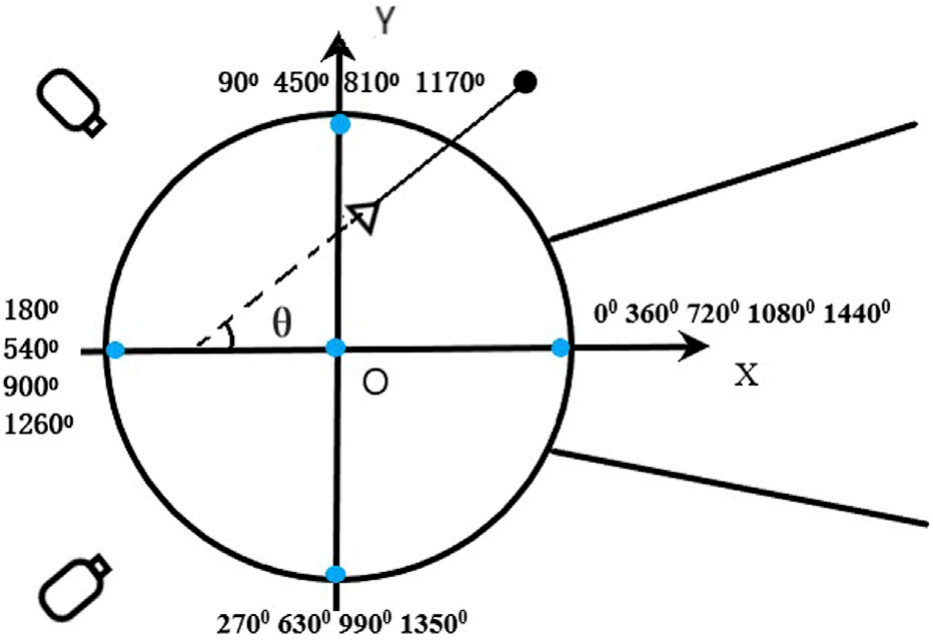
θ is the hammer horizontal azimuth angle.

The data of this study were collected from the 2022, 2023, and 2024 China Athletics World Championships Trials, the China Athletics Open, and the National Championships. Two high-speed cameras (ZcamE2, Shenzhen Vision Technology Co., Ltd.) were set at a height of 1.5 m, were connected using a synchronization cable, and were positioned on the right and left side of the throwing circle, respectively, with the 90-degree optical axes of the two video camcorders and 5–6 m horizontally distance from the center of throwing circle (Figure 1). All trials were recorded at 60 frames per second, a shutter speed of 1/1,600 s, and 1,920 × 1,080 resolution. Before the competition, a 28-point calibration frame (2.5 m long, 2 m wide, 2.5 m high) was used to calibrate camera positions and orientations. During calibration, five global reference frame markers were placed around the throwing circle to establish a global reference frame for data reduction.

For each trial, the two-dimensional coordinates of 21 body landmarks, the center of the hammerhead, and the intersection point of the handle and chain were manually digitized from video images. The digitization of each trial started five frames before the athlete’s right foot first left the ground and ended five frames after the hammer was released. The 3D positions of 21 body landmarks and two specific points on the hammer were calculated from synchronized 2D data using the direct linear transformation technique (Abdel-Aziz et al., 2015). The mean calibration error for each calibration was under 10 mm, with the maximum error not exceeding 10 mm. These coordinates were then converted into the global reference frame, with the *x*-axis pointing in the throwing direction, the *y*-axis pointing to the athlete’s left when facing the throwing direction, and the *z*-axis pointing upward (Figure 1). This conversion provided a detailed spatial representation of the athlete and equipment. The calculation of the 3D positions was performed using the digital processing software Fastmove Pose Creator (Dalian Sharp Motion Technology Co., Ltd.), and the 3D coordinates were then filtered using a Butterworth low-pass digital filter with an estimated optimal cutoff frequency of 7.14 Hz (Yu et al., 1999). The filtering was done using MATLAB R2022b.

### Data reduction

This study used the velocity of the center of the hammer head to represent the hammer’s velocity. Although there is a difference between the two, both reflect the athlete’s acceleration performance. The velocity-time curve of the hammer head’s center was obtained by calculating the first-time derivative of the smoothed coordinate-time curve using the central numerical differentiation method (Equation 1).

$$v_{x,n}=\frac{x_{n+1}-x_{n-1}}{2}\times F_{s}$$

$$v_{y,n}=\frac{y_{n+1}-y_{n-1}}{2}\times F_{s}$$

$$v_{z,n}=\frac{z_{n+1}-z_{n-1}}{2}\times F_{s}$$ (1)

Where $x_{n-1}$ , $y_{n-1}$ , $z_{n-1}$ are the coordinates of the hammer head’s center at the point immediately before the sampling point n ; $x_{n+1}$ , $y_{n+1}$ , $z_{n+1}$ are the coordinates of the hammer head’s center at the point immediately after the sampling point n ; $F_{s}$ is the sampling frequency (60fps). The resultant velocity of the hammerhead at the given sampling point n is (Equation 2):

$$v_{n}=\sqrt{v_{x,n}^{2}+v_{y,n}^{2}+v_{z,n}^{2}}$$ (2)

Hammer velocity increment: The hammer velocity increment for a given phase is defined as the final velocity at the end of the phase minus the initial velocity at the beginning.

Hammer horizontal azimuth angle: The vector from the intersection of the hammer handle and chain towards the center of the hammerhead is projected onto the X-O-Y plane of the global reference frame. The angle formed between the projected vector and the negative *Y*-axis is the horizontal azimuth angle of the hammer, which is the θ angle shown in Figure 1.

Hammer rotation radius: In this study, the hammer’s rotation radius in this study was calculated as the distance from the center of the hammer head to the midpoint of the line connecting the centers of both shoulders. Research indicates that trunk posture has a direct impact on the rotation radius. A 1-degree backward trunk tilt reduces the hammer rotation radius by approximately 4.6 mm (Dapena and Mcdonald, 1989). The calculations for the data above were performed using MATLAB R2022b.

### Data analysis

Based on the right foot’s takeoff and touchdown, the turns and delivery were divided into single support (SS) and double support (DS) phases. Each of the first three turns includes four critical instants: 1) right foot takeoff (Ron); 2) hammer head’s highest point (HP); 3) right foot touchdown (Roff); and 4) hammer head’s lowest point (LP). In the fourth turn, the point of release (Rel) is also added. The highest and lowest points of the hammerhead are defined as the positions of the highest and lowest points in each turn.

### Statistical procedures

Paired sample t-tests were used to compare the differences between the longest and the shortest trials in terms of velocity increments at various stages, the duration of single/double support phases, the horizontal azimuth angle of the hammer, and the rotation radius of the hammer at critical instants.

Statistical analyses were performed using IBM SPSS Statistics 27. To indicate statistical significance, a Type I error rate of less than or equal to 0.05 was chosen.

## Results

### The average throw distance

Table 1 presents the average throwing distance for the selected trials, revealing a significant difference between the two trials (*p* < 0.001, ES = 3.28).

**TABLE 1:** Means and standard deviations of official distance.

Trial	Official distance
Long (m)	62.404 ± 6.153
Short (m)	57.111 ± 5.821
*P-value*	<0.001
Cohen’s d	3.277

Long, the long distance trials.

Short, the short distance trials.

### Difference in hammer speed

**FIGURE 2:**
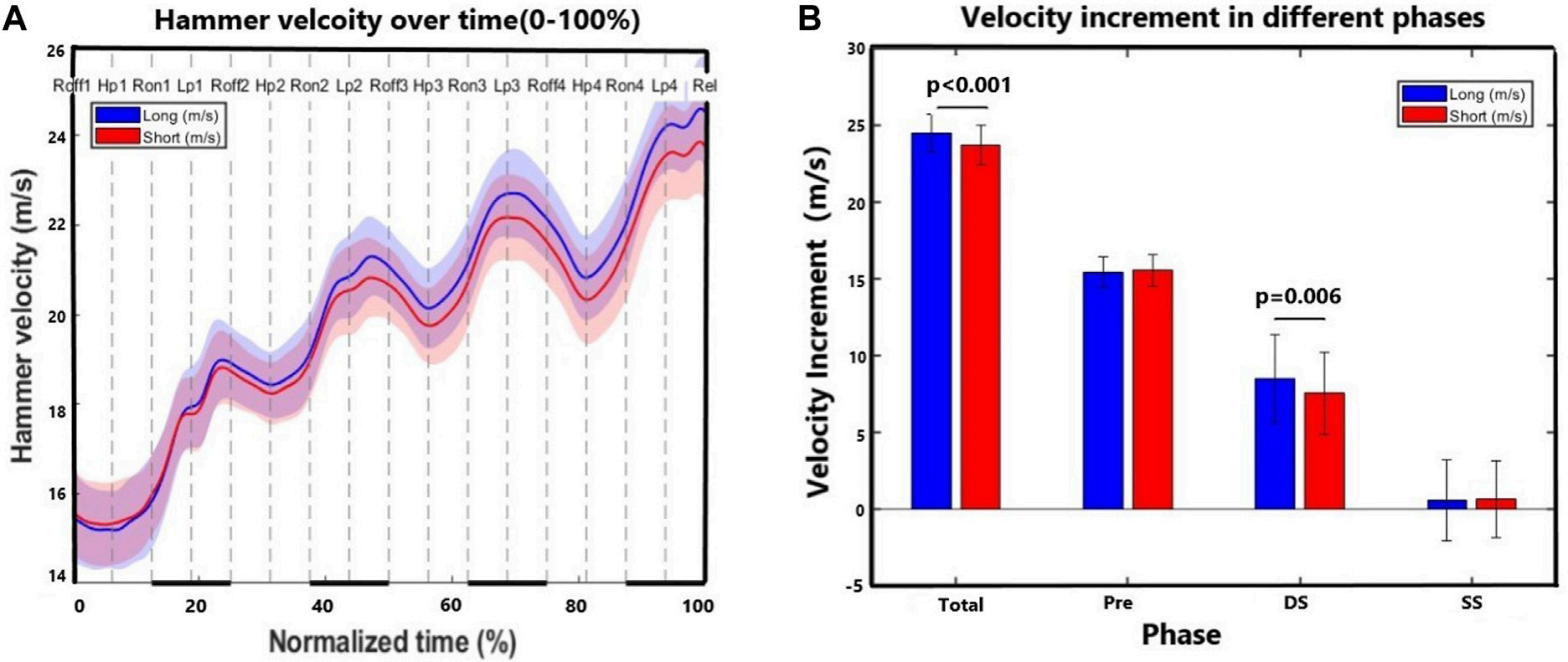
In **(A)**,the vertical axis represents the hammer’s velocity, while the horizontal axis represents the normalized time (0%–100%), with 0 indicating the velocity increment during the preliminary wind phase (the right foot first takes off the ground). The thick lines on the horizontal axis denote the double support phases, and the thin lines denote the single support phases. In **(B)**, “Total” indicates the total velocity increment during the entire phase, which is also the hammer release velocity. (Total = Pre + DS + SS); “Pre” indicate velocity increment during the preliminary wind phase; “DS” indicate the sum of velocity increments during the four double support phases; “SS” indicate the sum of velocity increments during the four single support phases.

Figure 2A shows the hammer velocity curve from the instant the right foot first takes off to the instant the hammer is released. It demonstrates that the hammer velocity curves of the two trials began to diverge following the first turn. Figure 2B shows a statistically significant difference (*p* < 0.05, ES = 1.42) was observed in the hammer velocity at the instant of hammer release and in the total velocity increment during the double support phase. A non-significant difference (*p* > 0.05, ES = 0.06–0.24) was observed in velocity increment during the preliminary wind and the total single support phase between the long and short trials.

### Difference in hammer rotation duration

Table 2 presents the differences in rotation duration between single and double support phases in each turn. There were no significant differences in the duration during the double support phase in any turn between the long and short trials (*p* > 0.05, ES = 0.08–0.23). The long trials had a significant (*p* < 0.05, ES = 0.42–0.83) shorter duration in the 1^st^ and 4^th^ turn and the total of single support phases, compared to the shorter trials.

**TABLE 2:** Duration in the single and double support phases for each rotation

Phase	Trial	1st turn	2nd turn	3rd turn	4th turn	Sum
SS (s)	Long	0.306 ± 0.033	0.251 ± 0.027	0.235 ± 0.023	0.231 ± 0.023	1.037 ± 0.081
	Short	0.314 ± 0.035	0.257 ± 0.019	0.241 ± 0.022	0.239 ± 0.024	1.103 ± 0.080
	*P*-value	**0.038**	0.142	0.106	**0.043**	<**0.001**
	*Cohen’s d*	0.429	0.297	0.329	0.418	0.834
DS (s)	Long	0.339 ± 0.038	0.265 ± 0.024	0.240 ± 0.029	0.255 ± 0.026	1.099 ± 0.082
	Short	0.335 ± 0.035	0.272 ± 0.030	0.242 ± 0.028	0.258 ± 0.029	1.106 ± 0.078
	*P*-value	0.433	0.259	0.694	0.395	0.478
	*Cohen’s d*	0.156	0.226	0.078	0.17	0.141

SS, the single support phase; DS, the double support phase.

Bold values means Significant Difference.

### Difference in hammer horizontal azimuth angle

Table 3 displays the differences in the horizontal azimuth angle of the hammer at critical instants during the 1st to the 4th turns. The non-significant difference was observed in the horizontal azimuth angle between long and short trials at the right foot takeoff, the highest point, and the lowest point of the hammerhead (*p* > 0.05, ES = 0.06–0.36). At the right foot touchdown, the long trials had a significantly lower angle in the 2nd, 3rd, and 4th turns compared to the short trials (*p* < 0.05, ES = 0.46–0.57).

**TABLE 3:** Horizontal azimuth angle at critical instants for each turn.

	Trial	1st turn	2nd turn	3rd turn	4th turn
Right foot takeoff (^o^)	Long	−7.683 ± 22.750	361.412 ± 20.728	718.373 ± 20.579	1,078.157 ± 20.547
	Short	−8.228 ± 23.534	363.961 ± 19.940	723.142 ± 23.030	1,082.967 ± 24.058
	*P*-value	0.717	0.217	0.110	0.077
	*Cohen’s d*	0.072	0.248	0.325	0.361
High point (^o^)	Long	66.172 ± 15.784	438.255 ± 11.699	807.894 ± 10.719	1,175.025 ± 10.519
	Short	68.198 ± 14.707	439.839 ± 13.730	809.133 ± 14.692	1,174.101 ± 14.588
	*P*-value	0.092	0.263	0.449	0.576
	*Cohen’s d*	0.344	0.225	0.151	0.111
Right foot Touchdown (^o^)	Long	147.900 ± 23.362	520.539 ± 13.426	884.641 ± 18.199	1,249.560 ± 15.248
	Short	151.840 ± 23.925	524.938 ± 16.577	891.625 ± 17.867	1,255.642 ± 20.754
	*P*-value	0.054	**0.020**	**0.008**	**0.027**
	*Cohen’s d*	0.397	0.486	0.565	0.462
Low point (^o^)	Long	250.350 ± 14.138	623.964 ± 11.171	992.250 ± 10.913	1,354.978 ± 10.582
	Short	251.538 ± 14.445	624.904 ± 12.817	994.861 ± 10.947	1,354.301 ± 14.333
	*P*-value	0.362	0.623	0.195	0.782
	*Cohen’s d*	0.182	0.098	0.261	0.055

Bold values means Significant Difference.

### Difference in hammer rotation radius

Table 4 shows the differences in the hammer rotation radius at critical instants from the 1st to the 4th turns. At the right foot takeoff, the hammer rotation radius of the long trials was significantly larger in the 2nd turn compared to the short trials (*p* < 0.05, ES = 0.48). At the highest point of the hammerhead, the hammer rotation radius in the long trials was significantly larger from the 1st to the 3rd turns compared to the short trials (*p* < 0.05, ES = 0.52–0.73). At the right foot touchdown, the hammer rotation radius in the long trials was significantly larger in the 2nd turn compared to the short trials (*p* < 0.05, ES = 0.51). There was no significant difference in the hammer rotation radius at the lowest point of the hammer head between the long and short trials (*p* > 0.05, ES = 0.05–0.21).

**TABLE 4:** Hammer rotation radius at critical instants for each rotation.

	Trial	1st turn	2nd turn	3rd turn	4th tun
Right foot takeoff (m)	Long	1.786 ± 0.040	1.804 ± 0.040	1.794 ± 0.038	1.796 ± 0.038
	Short	1.777 ± 0.037	1.789 ± 0.035	1.794 ± 0.038	1.789 ± 0.033
	*P-value*	0.170	**0.021**	0.948	0.108
	*Cohen’s d*	0.277	0.482	0.013	0.327
High point (m)	Long	1.801 ± 0.046	1.792 ± 0.039	1.772 ± 0.040	1.760 ± 0.040
	Short	1.787 ± 0.037	1.772 ± 0.035	1.760 ± 0.037	1.749 ± 0.040
	*P-value*	**0.003**	**0.001**	**0.013**	0.176
	*Cohen’s d*	0.565	0.728	0.524	0.273
Right foot touchdown (m)	Long	1.765 ± 0.043	1.770 ± 0.035	1.764 ± 0.037	1.763 ± 0.041
	Short	1.760 ± 0.043	1.757 ± 0.038	1.758 ± 0.032	1.758 ± 0.038
	*P-value*	0.227	**0.015**	0.219	0.384
	*Cohen’s d*	0.203	0.512	0.247	0.174
Low point (m)	Long	1.710 ± 0.039	1.734 ± 0.040	1.748 ± 0.041	1.754 ± 0.046
	Short	1.705 ± 0.045	1.732 ± 0.039	1.746 ± 0.041	1.753 ± 0.042
	*P-value*	0.299	0.777	0.614	0.799
	*Cohen’s d*	0.208	0.056	0.100	0.050

Bold values means Significant Difference.

## Discussion

This study employed a distinctive design. Each participant’s longest and shortest trials were selected from the same competition, typically occurring within an hour. This strategy aimed to minimize the influence of individual physical conditions and environmental factors on their performance. Consequently, the differences in official distance between the long and short trials observed in this study are primarily attributed to variations in the technique (Liu and Yu, 2022). The statistically significant differences in this study reflect a general pattern for the given variable between long and short trials among most participants.

We found that the difference in release speed between long and short trials was primarily attributed to the velocity increment during the double support phase rather than the preliminary wind and single support phase, supporting our 1st hypothesis. Although the velocity increment during the preliminary wind constitutes the largest proportion of the hammer throw release velocity, and many technical articles on hammer throwing highlight the crucial role of the preliminary wind on a successful throw (Gutienez-Davila and Rojas-Ruiz, 2005; Kelley, 2014; Rozhkov et al., 2020), this study did not observe differences in velocity increment between the two trials in this stage. There might be two reasons for this. Firstly, the trials we selected were performed by the same athlete within the same competition. Athletes tend to maintain a relatively stable speed rhythm during the preliminary wind when their physical condition remains unchanged. Secondly, athletes maintain a stable stance with both feet in contact with the ground during this stage, and the velocity of the hammer is relatively slow, making it easier to control (Hay, 1978). Thus, even if there are technical deviations between the long and the short trials, athletes are able to accelerate the hammer. Once entering the turning stage, the increase in hammer velocity and the instability of body posture during the single support phase make it more difficult for athletes to control both their body and the hammer (Castaldi et al., 2022). Technical issues arising during the preliminary wind can easily affect the acceleration of the hammer during the double support phase, thereby diminishing its acceleration effect. The lack of significant differences in velocity increment during the single support phase may be related to the technical objective of this phase, which is to “surpass the equipment” in preparation for accelerating the hammer during the double support phase (Bartonietz, 2000). Hence, it becomes evident that when an athlete’s physical condition remains unchanged, ensuring a velocity increment during the double support phase is critical to maximizing their performance. From the analysis above, enhancing the hammer’s release velocity can be understood as increasing the velocity increment during the double support phase.

The results do not support our 2nd hypothesis totally, given that the duration of the double support phase did not differ significantly between the long and short trials, whereby the single support phase was indeed considerably shorter in the long trials. According to the momentum principle, we initially assumed that a longer duration of the double support phase would benefit the hammer’s acceleration. However, our findings indicate that the duration of the double support phase may be a minor factor contributing to the discrepancy in distance between short and long trials. This may be due to the difficulty in further accelerating the hammer after the hammerhead passes the lowest point in each turn, as the parallel alignment of the shoulder and hip axes makes additional acceleration challenging (Brice et al., 2018). The hammer velocity curves also show that the hammer begins to decelerate from the low point after the second turn (Figure 2A). Therefore, deliberately extending the duration of the double support phase is of limited significance. Intriguingly, previous findings have shown that the muscle power from the upper limbs was not related to performance in hammer throw analyzing athletes from different athletic modalities (Zhao et al., 2023). Our results highlight the importance of analyzing the performance specifically in athletes from the same modality to get deeper insights into the performance determinants.

The results support our 3^rd^ hypothesis that the horizontal azimuth angle of the hammer is smaller at critical instants (only at the right foot touchdown) in the long trials compared to the short trials. At the beginning of the double support phase, forming a maximally “wound-up” posture (with the hammer trailing far behind the feet, hips, and shoulders) is essential for effectively utilizing body power to accelerate the hammer (Hay, 1978). In this study, we define the horizontal azimuth angle of the hammer as the angle between the projection of the hammer in the horizontal plane and the negative *y*-axis. This angle gradually increases from the right foot takeoff to the right foot touchdown. A smaller azimuth angle at the right foot touchdown indicates that the hammer is more delayed in its spatial position (closer to the hammer’s high point), which may be more conducive to forming a wound-up posture where the body surpasses the hammer. Additionally, a smaller azimuth angle of the hammer at the right foot touchdown may also help extend the azimuthal angle covered by the hammer during the double support phase. Some researchers believe that expanding the range of azimuth angle covered by the hammer during the double support phase, rather than the time spent, is key to enhancing the velocity increment of the hammer during this phase (Sedykh and Strelnitski, 2018).

The results support our 4th hypothesis that the radius of the hammer rotation is larger at the critical instants in the long trials compared to the short trials. In the previous paragraph, we discussed the advantages of a smaller hammer azimuth angle at the right foot touchdown for increasing the velocity increment during the double support phase. Athletes need to pay particular attention to certain technical aspects during the single support phase to achieve a smaller azimuth angle at this critical instant (Hay, 1978). As the hammer’s horizontal azimuth angle increases continuously from the right foot takeoff to touchdown (during the single support phase), the horizontal azimuth angle of the hammer at the right foot touchdown is influenced by two factors: 1) The right foot landing speed: The shorter the time between the right foot takeoff and touchdown, the more the hammer head may lags relative to the body, which helps to reduce the hammer’s horizontal azimuth angle. Table 2 shows that the long trials have a significantly shorter duration in the single support phase of the first and fourth turn, as well as a significantly shorter total single support duration, compared to the short trials; 2) The rotational radius of the hammer head during the single support phase: when the linear velocity remains constant, a larger rotational radius of the hammer head results in a lower angular velocity (Dapena, 1984), which may also cause the hammer head to lag more relative to the body at the instants of right foot touchdown. Table 4 shows that the rotational radius of the hammerhead at the instants of the right foot taking off and the hammerhead’s highest point is significantly larger in the long trials compared to the short trials.

In comparing the difference in techniques between long and short trials, although differences in the velocity increment occurred during the double support phases, the single support phase indirectly influences the hammer head’s acceleration performance during the double support phase. This reaffirms the primary technical objective of the single support phase to create space for accelerating the hammer during the double support phase. These findings suggest that athletes’ shoulders should be relaxed and pulled well forward from the hammer head’s lowest points to the instants the right foot taking off (Hay, 1978), with the torso slightly leaning towards the hammer to avoid sudden backward force applied through the shoulder and hip axes. Upon entering the single support phases, athletes should maintain their right foot close to the ground, move it alongside the left leg to reduce rotational inertia, and quickly land it.

One of the limitations of this study is the low sampling frequency, and the common differences in body movements among different throwers are still not fully understood. Further studies should employ within-subject designs with relatively large sample sizes to identify discrepancies in body characteristics. This will provide additional insights to improve understanding of hammer throwing techniques. In addition, we did not combine male athletes, who may have different biomechanics characteristics from female athletes (Konz and Hunter, 2015). Future research could compare male and female athletes to get a deeper insight into hammer throwing.

## Conclusion

During the process from the hammer head’s lowest to highest points, athletes should focus on increasing the hammer head’s rotational radius and accelerating the right foot’s landing speed during the single support phase. This approach aims to reduce the hammer’s horizontal azimuth angle at the right foot touchdown, enhance the acceleration performance during the double support phase, and increase the release speed.

## Data Availability

The original contributions presented in the study are included in the article/supplementary material, further inquiries can be directed to the corresponding author.
